# Optimizing heartworm diagnosis in dogs using multiple test combinations

**DOI:** 10.1186/s13071-021-04715-4

**Published:** 2021-04-26

**Authors:** Jennifer N. Lane, Annette Litster, Susan E. Little, Jessica Y. Rodriguez, Kennedy K. Mwacalimba, Kellee D. Sundstrom, E. Susan Amirian, Simone D. Guerios, Maria A. Serrano, Kellie M. Hays, Julie K. Levy

**Affiliations:** 1 Maddie’s Shelter Medicine Program, College of Veterinary Medicine, University of Florida, 2015 SW 16th Avenue, Gainesville, FL 32610 USA; 2 Zoetis Petcare, 10 Sylvan Way, Parsippany, NJ 07054 USA; 3 Department of Veterinary Pathobiology, Oklahoma State University, 250 McElroy Hall, Stillwater, OK 74078 USA; 4 Research Department, Austin Pets Alive!, Austin, TX 78703 USA; 5 Department of Small Animal Clinical Sciences, College of Veterinary Medicine, University of Florida, 2015 SW 16th Avenue, Gainesville, FL 32610 USA

**Keywords:** Antibody, Antigen, Diagnosis, Heartworm, Microfilaria

## Abstract

**Background:**

Various heartworm (HW) diagnostic testing modalities detect products of, or reactions to, different life cycle stages of *Dirofilaria immitis*. Microfilariae (Mf) can be directly visualized in blood, antigen (Ag) from immature and adult heartworms may be detected on commercial assays, and antibody (Ab) tests detect the host immune response to larval stages. Ag and Mf tests are commonly used in dogs, which frequently carry adult HW infections, but Ab tests have only been validated for use in cats. In some HW-infected dogs, Ag is blocked by immune complexing leading to false-negative results. Heat-treatment (HT) to disrupt these complexes can increase the sensitivity of HW Ag tests. The aim of this study was to compare different methods for diagnosing HW infection in dogs at high risk using individual and paired diagnostic tests, including an exploration of using Ab tests designed for cats to test canine samples.

**Methods:**

One hundred stray adult (≥ 2-year-old) dogs in Florida shelters were tested using Mf, HW Ag, and HW Ab tests (feline HW Ab tests currently not commercially validated/approved for use in dogs); two versions of each test platform were used.

**Results:**

Fourteen dogs tested positive using point-of-care (POC) Ag tests; an additional 2 dogs tested positive with microtiter well assay, and an additional 12 dogs tested positive using HT Ag testing. For individual tests, Ag test sensitivity/specificity compared to HT Ag was 50–57%/100%, and Ab tests were 46–64%/82–94%. Sensitivity estimates for individual tests were higher when comparing to non-HT Ag. Pairing POC Ag tests with Mf tests improved sensitivity without loss of specificity, while pairing POC Ag and Ab tests modestly increased sensitivity at the expense of specificity.

**Conclusions:**

Screening dogs for HW infection using both POC Ag and Mf detection, which is recommended by the American Heartworm Society, improved diagnostic performance in this study compared to single Ag test use, but may have missed more than one in four infected dogs. The need to improve access to highly accurate, rapid, and inexpensive large-scale HW testing for dogs in animal shelters remains largely unmet by current testing availability. The development of practical and validated protocols that incorporate heat or chemical treatment to disrupt Ag-Ab complexes in POC testing or decreasing the cost and time required for such testing in reference laboratories might provide solutions to this unmet need. Similar studies performed in countries where the prevalence of parasites such as *D. repens* or *A. vasorum* is different to the USA could potentially yield very different positive predictive values for both HT and non-HT Ag tests.

**Graphic abstract:**

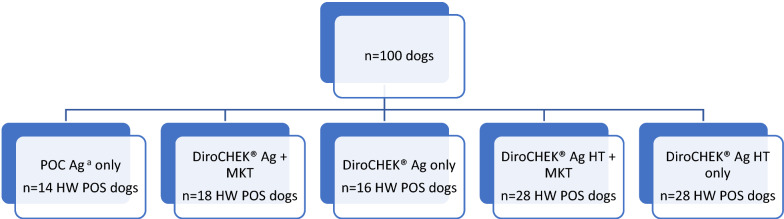

## Highlights

- Heartworm antigen (Ag), antibody (Ab), and microfilaria (Mf) test results from 100 stray dogs presented to Florida shelters were compared.
- Heat treatment (HT) of serum substantially increased HW Ag test positive results compared to point of care (POC) Ag tests.
- Mf were identified in 16 dogs; 13 *D. immitis*, 4 *Acanthocheilonema reconditum*, and 1 *Dirofilaria* sp. (sequenced as *D. repens*).

## Background

*Dirofilaria immitis* is the nematode responsible for canine heartworm (HW) disease and is transmitted by mosquitoes. This parasite infects the pulmonary arteries and heart, causing inflammation, endothelial proliferation, and pulmonary hypertension [25]. Clinical signs include cardiac murmurs, lethargy, exercise intolerance, and coughing, and many dogs progress to fatal pulmonary thromboembolism or cardiopulmonary failure. After initial mosquito transmission of third-stage larvae and migration of third- and fourth-stage larvae through connective tissues, young adult filariae can be found in the small distal pulmonary arteries and can then grow to up to 30 cm in length, occupying larger pulmonary arteries and, as worm numbers increase, the right ventricle. Sexual reproduction takes place in mature adult heartworms to produce microfilariae, which are the source of infection for mosquito vectors [25].

Published studies of currently available heartworm antigen tests report varying relative sensitivities and specificities [2, 8, 13, 16, 34]. From a practical perspective, failure to detect infected dogs can leave them at risk of ongoing disease, and incorrectly identifying infections can lead to unnecessary treatments. A multi-modal approach to diagnosis is thought to increase the likelihood of detection. Veterinary advisory boards recommend annual blood testing for *D. immitis* antigen (Ag) and microfilaria (Mf) in all dogs that live in areas where mosquito vectors exist [25]. Point-of-care (POC) Ag test platforms include enzyme-linked immunosorbent assay (ELISA) and rapid immunomigration (RIM) tests, which both detect Ag produced primarily by adult female worms [2]. In a study using necropsy-confirmed mature female HW infections from dogs naturally infected in the southeastern US state of Florida to compare POC Ag HW tests in dogs, sensitivities ranged from 97.5–99.5% and specificities were 94% with an overall 98.4–99.2% agreement with DiroCHEK [16]. In another study evaluating naturally infected dogs in the same region, sensitivities of DiroCHEK for all infections (immature and mature), mature worms (male and female), and mature female worms were 86.9%, 90.7%, and 93.3%, respectively. Dogs with one mature worm present were detected as antigen-positive in 55.6% of cases and 90.0% of dogs with two worms were antigen-positive; however, 75% of dogs with 3–5 worms were antigen-positive [13].

Circulating Ag can become trapped in immune complexes in both dogs and cats, rendering Ag undetectable and leading to false-negative results [23]. Heat treatment (HT) or chemical treatment to destroy complexes can increase Ag test sensitivity [4, 9, 22, 23]. However, HT protocols require extra laboratory equipment, time, and cost, reducing utility when large numbers of dogs are tested on a daily basis, such as in animal shelters in HW-endemic regions. While HT has value in the investigation of dogs suspect for HW infection on clinical grounds, dogs with Ag-negative results, and dogs that test Ag-negative but Mf-positive, this approach is not currently recommended for routine screening for canine HW infection [23]. While diagnostic laboratories offer HT HW Ag testing, the associated cost and delay in receiving results could present implementation challenges for many shelters.

In cats, HW antibody (Ab) testing detects Abs produced in response to third- and fourth-stage larvae and young adult infections [26]. HW antibodies have been detected in experimentally infected dogs [18], but HW Ab tests are currently not commercially validated/approved for use in dogs.

Heartworm prevalence in the USA appears to be increasing, and dogs presented to animal shelters are a particularly high-risk group [9, 10–12, 15, 17, 35]. Municipal animal shelters tasked with housing and caring for large numbers of stray or unowned dogs often struggle to have adequate resources, time, and expertise to prevent, diagnose, and treat HW infection [7, 29]. More practical and accurate screening procedures are needed for dogs entering shelters, so that individual dogs and populations can be managed effectively. Combining two tests may increase overall diagnostic performance in dogs, as has been reported in cats [3, 32]. Since HW Ab testing primarily detects the immune response to an earlier stage of the parasitic life cycle than Ag testing and can detect male and single-sex infections, this approach could potentially enhance detection in dogs. The aim of this study was to compare methods used to diagnose HW infection in dogs at high risk using individual and paired diagnostic tests, including an exploration of the use of Ab tests designed for use in cats.

## Methods

Data reported here were collected in conjunction with research comparing heartworm prevalence in geographically and age-matched shelter dogs and cats [15].

This study enrolled 100 stray dogs estimated to be 2 years or older (based on dental examination—all teeth were fully erupted, yellowed, and carried tartar; Humane Society of the United States [19]). All dogs were newly admitted to one of three Florida animal shelters and with no history of macrocyclic lactone administration. Forty-nine dogs were sampled at a shelter located in north-central Florida (Marion County and Columbia County), and 51 dogs were sampled at a shelter in south Florida (Miami-Dade County). All dogs were sourced locally. Blood sample collection occurred between May and June 2019, during routine health examinations.

Approximately 6–7 ml of blood was collected from each dog. From each specimen, a 2 ml aliquot was placed in an EDTA tube and 4–5 ml was placed in a serum separator tube (SST). The SST was allowed to clot at room temperature, followed by centrifugation to separate the serum. Serum aliquots were frozen at –20 °C pending analysis.

On the day of collection, fresh whole blood from the EDTA tube was tested for *D. immitis* Ag using a POC assay WITNESS® Heartworm antigen test (WHW; Zoetis LLC, Parsippany, NJ). Serum was tested for HW antigen by another POC, SNAP® Heartworm RT Test (SNAP; IDEXX Laboratories, Inc., Westbrook, ME, USA). Within 2 weeks, serum was tested for HW Ag using the ELISA test DiroCHEK® (DHW; Zoetis LLC, Parsippany, NJ, USA) before and after HT, as previously described [22, 23].

Samples were refrigerated pending Mf testing within 4 days of collection. Ten microliters of blood was mixed with 10 µl of saline to create a wet mount (WM) on a glass slide, which was examined microscopically at 10× and 40× for Mf. No attempt was made to determine the identity of the motile Mf on WM. A modified Knott's test (MKT) was performed to: (1) quantitate Mf present and (2) identify Mf using morphological characteristics. The test was considered positive for Mf if any Mf were seen and positive for *D. immitis* if morphology and size were consistent with *D. immitis* standards [24]. Because of the potential for other filarial species, we only identified *D. immitis* and *A. reconditum* by morphology. Large filarial species were identified as *Dirofilaria* sp.

PCR was used to identify microfilariae in frozen blood samples, and sequencing was conducted as previously described [28]. Briefly, DNA was extracted from 200 µl of whole blood using a commercial kit (DNeasy Blood Kit; Qiagen, Hilden, Germany) and served as template in a PCR targeting a ~ 330 bp fragment of the 12S rRNA gene. Electrophoresis on a 2% agarose gel was used to confirm amplification. Amplicons were column-purified, and an ABI 3730 capillary sequencer was used for sequencing (Applied Biosystems; Foster City, CA, USA) at the Oklahoma State University Molecular Core Facility (Stillwater, OK, USA). Visual examination of electropherograms was conducted, comparing to all available sequences in GenBank (National Center for Biotechnology Information) with the GenBank accession numbers of the closest matching sequences reported.

Serum was tested for HW antibodies using two tests developed for use in cats but not validated for canine serum: (1) a POC test (SSA; Solo Step® FH, Heska, Loveland, CO, USA) and (2) an ELISA test (ANT; Antech Feline Heartworm Antibody test, Antech Diagnostics, Fountain Valley, CA, USA). These tests are not approved for use in species other than cats. Their use in this study was purely to investigate their potential to improve the detection of canine heartworm infection in shelter dogs.

Diagnostic assays were performed by separate teams, each masked to the other’s results. Ag testing with the WHW POC test was performed at each shelter by the University of Florida research team. SNAP, DHW, SSA, WM, MKT, and submission of serum for ANT were performed by the Oklahoma State University Veterinary Parasitology research team in Stillwater, Oklahoma. Another individual at Oklahoma State University performed microfilariae PCR and analyzed sequence data obtained through an academic core facility.

Dogs were considered infected with adult *D. immitis* based on a positive result on at least one of the three *D. immitis* Ag tests or identification of *D. immitis* Mf confirmed by morphology and/or PCR and sequencing. Dogs that were positive on Ab test, but not on any other assays, were not considered infected with *D. immitis*.

Due to the lack of a standard for case ascertainment, two different sensitivity and specificity calculations were made, one using DHW following HT of serum and the other using DHW only (MedCalc). HT of serum may maximize sensitivity and potentially reduce specificity [23]; therefore, examining the differences in test operating characteristics compared to DHW with HT and non-HT serum can be informative.

Test operating characteristics were also calculated for pairs of Ag and Ab tests, relative to the aforementioned reference standards (i.e., DHW with HT and non-HT serum). Each pair of tests was considered positive for HW if at least one of the two tests yielded a positive result. It was considered negative if both tests were negative. This strategy was employed to maximize sensitivity over specificity.

## Results

The performance of all individual assays compared to DHW both without and with heat treatment of serum is reported in Table 1. Detailed results for the 42 dogs that had at least one positive test result are shown in Table 2. Regarding HW Ag testing, DHW HT results were positive in 28 dogs, including all dogs with at least one positive POC Ag test result (*n* = 14) and all dogs positive on DHW before HT (*n* = 16; this includes all 14 dogs with at least one positive POC Ag test result). An additional 12 dogs had DHW HT-positive, but POC and DHW Ag-negative results.

**Table 1:** Sensitivity and specificity of diagnostic tests in 100 stray adult dogs in Florida animal shelters compared to heartworm antigen (Ag) detection in (a) non-heat-treated serum and (b) heat-treated serum

Tests	Sensitivity (%)	Sensitivity 95% CI (%)	Specificity (%)	Specificity 95% CI (%)	Total positive results (*n*)	False-negative results (*n*)	False-positive results (*n*)
a. Test operating characteristics compared to *non-heat-treated* serum with the DiroCHEK® Antigen test. A total of 16 dogs had positive results with non-heat-treated serum							
WITNESS® HW Ag	88	62–98	100	96–100	14	2	0
SNAP® Ag	88	62–98	100	96–100	14	2	0
DiroCHEK® Ag (heat treated)	100	79–100	86	76–92	28	0	12
HESKA Solostep® FH (Ab)	69	41–89	76	66–85	31	5	20
Antech heartworm antibody test	56	30–80	90	82–96	17	7	8
b. Test operating characteristics compared to *heat-treated* serum with the DiroCHEK® Antigen test. A total of 28 dogs had positive results with heat-treated serum							
WITNESS® HW Ag	50	31–69	100	95–100	14	14	0
SNAP® Ag	50	31–69	100	95–100	14	14	0
DiroCHEK® Ag (non-heat treated)	57	37–76	100	95–100	16	12	0
HESKA Solostep® FH (Ab)	64	44–81	82	71–90	31	10	13
Antech heartworm antibody test	46	28–66	94	86–98	17	15	4

Ab, antibody; Ag, antigen; CI, confidence intervals; HW, heartworm; WHW, WITNESS® Heartworm; DHW, DiroCHEK®; HT, heat-treated antigen test; SNAP, IDEXX SNAP® Heartworm RT; *D. immitis*, *Dirofilaria immitis*

^a^Relative to HT DHW

**Table 2:** Diagnostic test results for 42 Florida shelter dogs with a positive result on at least one heartworm test

Dog ID number	Point of care		Reference lab		Antibody		Microfilaria			
	WHW	SNAP	DHW	DHW HT	ANT	SSA	WM	MKT	Mf/ml	Sequence identity
D01	−	−	−	+	−	−	−	−	−	N/A
D02	−	−	−	+	−	−	+	+	142	*Acanthocheilonema reconditum*
D07	−	−	−	+	+	+	−	−	−	N/A
D08	+	+	+	+	+	−	−	−	−	N/A
D14	+	+	+	+	+	+	+	+	15,220	Poor quality^a^
D16	−	−	−	−	−	+	−	−	−	N/A
D22	−	−	+	+	−	−	+	+	47	*Acanthocheilonema reconditum*
D24	−	−	−	+	−	−	+	+	7	*Acanthocheilonema reconditum*
D25	−	−	−	−	−	+	−	−	−	N/A
D27	+	+	+	+	−	+	+	+	3400	*Dirofilaria immitis*
D28	+	+	+	+	+	+	+	+	58	*Dirofilaria immitis*
D31	−	−	−	+	+	+	+	+	1102	*Dirofilaria immitis*
D32	+	+	+	+	+	+	+	+	2050	*Dirofilaria immitis*
D34	+	+	+	+	−	+	+	+	3	No amplification^b^
D35	+	+	+	+	−	−	−	−	−	N/A
D39	−	−	−	−	−	+	−	−	−	N/A
D40	−	−	−	−	−	+	−	−	−	N/A
D41	−	−	−	−	−	+	−	−	−	N/A
D43	+	+	+	+	−	+	+	+	606	*Dirofilaria immitis*
D44	−	−	−	+	−	−	−	−	−	N/A
D45	−	−	−	−	−	+	−	−	−	N/A
D48	−	−	−	+	−	+	−	+	13	*Dirofilaria immitis*
D49	−	−	−	−	−	+	−	−	−	N/A
D53	+	+	+	+	−	−	+	+	11,740	*Dirofilaria immitis*
D54	−	−	−	−	−	+	−	−	−	N/A
D57	−	−	−	+	−	+	+	+^c^	158	*Dirofilaria repens*
D65	−	−	−	−	−	+	−	−	−	N/A
D68	−	−	−	+	+	+	−	−	−	N/A
D70	−	−	−	−	+	+	−	−	−	N/A
D77	−	−	−	+	+	+	−	−	−	N/A
D79	−	−	−	+	−	+	−	−	−	N/A
D81	+	+	+	+	−	−	+	+	8210	*Dirofilaria immitis*
D83	+	+	+	+	+	+	+	+	5250	*Dirofilaria immitis*
D84	−	−	−	−	+	+	−	−	−	N/A
D86	−	−	+	+	+	+	−	−	−	N/A
D87	+	+	+	+	+	+	+	+	180	*Dirofilaria immitis*
D90	+	+	+	+	+	+	+	+	43,280	*Dirofilaria immitis*
D93	−	−	−	−	−	+	−	−	−	N/A
D94	−	−	−	−	+	+	−	−	−	N/A
D96	−	−	−	+	−	−	−	−	−	N/A
D99	+	+	+	+	+	+	+	+	1293	*Dirofilaria immitis*
D100	−	−	−	−	+	−	−	−	−	N/A
*n* Positive for test	14	14	16	28	17	31	17	18		

ID, identification; −, negative; +, positive: N/A, not applicable; WHW, WITNESS® Heartworm; DHW, DiroCHEK®; HT, heat-treated antigen test; SNAP, IDEXX SNAP® Heartworm RT; ANT, Antech heartworm antibody test; SSA, HESKA SoloStep® FH; MKT, modified Knott’s test for *D. immitis*; Quantitative MKT, microfilaria/ml using MKT; WM, wet mount; *A. reconditum*, *Acanthocheilonema reconditum*; *D. immitis*, *Dirofilaria immitis*

^a^Multiple traces present, likely mixed population *D. immitis* microfilaria; *D. immitis* on Knott’s test morphology

^b^*A. reconditum* on Knott’s test morphology

^c^Morphology on MKT was *Dirofilaria* sp.; PCR and sequencing were performed to definitively determine species

Wet mount revealed Mf in 17 dogs, and MKT identified Mf in 18 dogs. Morphometric analysis of microfilariae recovered on MKT identified 13 as *D. immitis*, 4 as *Acanthocheilonema reconditum*, and 1 as a *Dirofilaria* sp. not consistent with those found in North America. The single dog positive on MKT but negative on WM had 13 *D. immitis* Mf/ml on MKT. Of the 18 dogs with Mf on MKT, PCR and sequencing confirmed 12 as *D. immitis,* 3 as *A. reconditum,* and 1 as *D. repens* (Table 2). Samples from two dogs with Mf on MKT, one with 3 *A. reconditum* Mf/ml and the other with > 15,000 *D. immits* Mf/ml, did not amplify or sequence (Table 2).

Of the 13 dogs with *D. immitis* Mf by MKT or PCR and sequencing, Ag was detected in 11 by POC, DHW, and DHW HT and in 2 by DHW HT only. Of the four dogs with *A. reconditum* Mf by MKT or PCR and sequencing, Ag was detected in two by POC and DHW HT and in two by DHW HT only. The one dog with Mf of *D. repens* by MKT and PCR and sequencing was positive on DHW HT but not POC tests (Table 2).

Antibody was detected in 33 dogs, including 15 with both SSA and ANT, 16 with SSA only, and 2 with ANT only (Table 2). Of the 31 dogs with antibodies detected on SSA, Ag was detected in 10 with POC tests, 11 with DHW, and 18 with DHW HT. Of the 17 dogs with antibodies detected on ANT, Ag was detected in 8 with POC tests, 9 with DHW, and 13 with DHW HT. Of the 13 dogs with *D. immitis* Mf on MKT, 11 were Ab positive on SSA and 8 were Ab positive on ANT.

Performing POC Ag testing alone resulted in the fewest dogs detected as positive. Combining WM with POC Ag testing resulted in five dogs that were POC Ag negative and Mf positive; MKT and sequencing confirmed that the Mf in those dogs were *A. reconditum* (*n* = 3), *D. immitis* (*n* = 1), and *D. repens* (*n* = 1). The addition of MKT to DHW revealed two additional HW infections (Ag negative and *D. immitis* Mf positive), both of which were also positive with DHW HT. (Table 3). Table 4 presents positive results for HW antigen (Ag) or antibody (Ab) in the context of their Mf status.

**Table 3:** Clinical heartworm test combinations and resulting number of dogs classified as heartworm-positive (*n* = 100 dogs)

Test/combination of tests	HW POS dogs	
POC Ag^a^ only		14
DiroCHEK® Ag + MKT		18
DiroCHEK® Ag only^b^		16
DiroCHEK® Ag HT + MKT		28
DiroCHEK® Ag HT only		28

*Ag* antigen, *HW* heartworm, *HT* heat treated, *MKT* modified Knott’s test, *POC* point of care test, *POS* positive

^a^Witness® and IDEXX SNAP® were performed with 100% agreement

^b^Of the 16 DiroCHEK® Ag-positive dogs, 13 were microfilaria-positive (*n* = 11 *D. immitis*; *n* = 2 *A. reconditum*, D22 and D34); the remaining 3 dogs were microfilaria-negative (D08, D35, and D86). An additional 5 dogs were DiroCHEK® Ag-negative and microfilaremic (*n* = 2 *D. immitis*, D 31 and D48; *n* = 2 *A. reconditum*, D02 and D24; *n* = 1 *D. repens*, D57)

**Table 4:** Dogs positive for heartworm antigen (Ag) or antibody (Ab) in context of their microfilariae (Mf) status

Mf results	POC^a^ Ag POS	DiroCHEK® Ag POS	DiroCHEK® Ag + HT POS	Ab POS^b^
All Mf NEG (*n* = 82)	2 (2.4%)	3 (3.7%)	10 (12.2%)	20 (24.4%)
*Di* Mf NEG (*n* = 87)	3 (3.4%)	5 (5.7%)	15 (17.2%)	22 (25.3%)
Any Mf POS (*n* = 18)	12(66.6%)	13 (72.2%)	18 (100%)	13 (72.2%)
*Di* Mf POS (*n* = 13)	11 (84.6%)	11 (84.6%)	13 (100%)	11 (84.6%)
*Dr* Mf POS (*n* = 1)	0 (0%)	0 (0%)	1 (100%)	1 (100%)
*Ar* Mf POS (*n* = 4)	1 (25%)	2 (50%)	4 (100%)	1 (25%)

POS, positive; NEG, negative; Ag, antigen; POC, point of care test: Witness and IDEXX SNAP were performed with 100% agreement; POS, positive; HT, heat treatment; *Di*, *Dirofilaria immitis*; *Dr*, *D. repens*; *Ar*, *Acanthocheilonema reconditum*

^a^Witness® and IDEXX SNAP® were performed with 100% agreement

^b^Heska and Antech Ab results were combined; these tests are not validated for use in dogs

Regarding test operating characteristics for pairs of Ag and Ab tests, when a HW Ab test was added to a single POC HW Ag test, sensitivity was modestly increased compared to results for the Ag test alone, but always at the expense of a reduction in specificity (Tables 1 and 5).

**Table 5:** Sensitivity and specificity of pairs of diagnostic tests in 100 stray adult dogs in Florida animal shelters compared to heartworm antigen (Ag) detection in (a) heat-treated serum and (b) non-heat-treated serum

Test Pairs	Sensitivity (%)	Sensitivity 95% CI (%)	Specificity (%)	Specificity 95% CI (%)	Total positive results (*n*)	False-negative results (*n*)	False-positive results (*n*)
a. Test operating characteristics compared to *heat-treated* serum with the DiroCHEK® Antigen test							
WITNESS® HW Ag & SNAP® Ag	50	31- 69	100	95–100	14	14	0
WITNESS® HW Ag & DiroCHEK® Ag (non-heat treated)	57	37–76	100	95–100	16	12	0
WITNESS® HW Ag & HESKA Solostep® FH (Ab)	79	59- 92	82	71–90	35	6	13
WITNESS® HW Ag & Antech heartworm antibody test	68	48–84	94	86–98	23	9	4
SNAP® Ag & DiroCHEK® Ag (non-HT)	57	37–76	100	95–100	16	12	0
SNAP® Ag & HESKA Solostep® FH (Ab)	79	59- 92	82	71–90	35	6	13
SNAP® Ag & Antech heartworm antibody test	68	48- 84	94	86–98	23	9	4
DiroCHEK® Ag (non-HT) & HESKA Solostep® FH (Ab)	82	63–94	82	71–90	36	5	13
DiroCHEK® Ag (non-HT) & Antech heartworm antibody test	71	51–87	94	86–98	24	8	4
HESKA Solostep® FH (Ab) & Antech heartworm antibody test	68	48–84	81	70–89	33	9	14
b. Test operating characteristics compared to *non-heat-treated* serum with the DiroCHEK® Antigen test							
WITNESS® HW Ag & SNAP® Ag	88	62–98	100	96–100	14	2	0
WITNESS® HW Ag & DiroCHEK® Ag (heat treated)	100	79–100	86	76–92	28	0	12
WITNESS® HW Ag & HESKA Solostep® FH (Ab)	94	70–100	76	66–85	35	1	20
WITNESS® HW Ag & Antech heartworm antibody test	94	70–100	90	82–95	23	1	8
SNAP® Ag & DiroCHEK® Ag (HT)	100	79–100	86	76–92	28	0	12
SNAP® Ag & HESKA Solostep® FH (Ab)	94	70–100	76	66–85	35	1	20
SNAP® Ag & Antech heartworm antibody test	94	70–100	90	82–96	23	1	8
DiroCHEK® Ag (HT) & HESKA Solostep® FH (Ab)	100	79–100	70	59–80	41	0	25
DiroCHEK® Ag (HT) & Antech heartworm antibody test	100	79–100	81	71–89	32	0	16
HESKA Solostep® FH (Ab) & Antech heartworm antibody test	75	48–93	75	64–84	33	4	21

Ab, antibody; Ag, antigen; CI, confidence intervals; HW, heartworm; WHW, WITNESS® Heartworm; DHW, DiroCHEK®; HT, heat-treated antigen test; SNAP, IDEXX SNAP® Heartworm RT; *D. immitis*, *Dirofilaria immitis*

## Discussion

Maximizing diagnostic accuracy is crucial when screening for canine HW infection. Failing to detect HW infection can lead to progression of disease or the transmission of infection to local mosquito populations [21]. Additional risks for animal shelters include the adoption of HW-infected dogs by unsuspecting families and the transport of infected dogs to regions with low HW prevalence where disease awareness is low [11]. Falsely identifying uninfected dogs as HW positive can lead to unnecessary treatment that results in extra cost, exercise restriction, and potential adverse events. Additionally, in an animal shelter environment, a false-positive HW diagnosis may also delay or prevent adoption. In this study, as well as POC Ag testing, we evaluated several testing modalities that are not typically performed when screening shelter dogs for HW (Mf testing, HT antigen testing, and heartworm antibody testing). Although we did estimate sensitivity and specificity using two different tests (DHW or DHW HT), this cannot represent the true diagnostic accuracy of these tests in this population, since we did not have necropsy confirmation of the presence or absence of heartworms.

The American Heartworm Society (AHS) does not currently recommend routine heat treatment of samples prior to HW Ag screening, as it is not consistent with the labeled protocol for licensed HW tests and may affect their accuracy, potentially decreasing the high specificity of HW antigen tests. The potential for HT leading to false-positive HW Ag tests by cross-reacting with other parasites has been evaluated in the field in naturally infected dogs. However, experimental infections, sampling in areas with no *D. immitis* transmission, and necropsy confirmatory studies using current commercial HW antigen tests only exist for *D. repens*, *A. vasorum,* and/or *A. reconditum* [14, 33, 36]*.* In three dogs experimentally infected with *D. repens,* all dogs converted from negative to positive post-HT using three commercial HW Ag tests [33]. Pairing these data with a field study investigating dogs naturally infected with *D. repens* in a region not endemic for *D. immitis* demonstrates the potential for cross-reactivity post-HT [36]. Based on these studies, it is likely that the one dog in our study that had *D. repens* Mf and converted from HW Ag negative to positive post-HT was a result of cross-reactivity; however, we cannot rule out the possibility that this dog was also infected with HW and the infection was identified post-HT, as co-infection with *D. repens* and *D. immitis* is common in areas where both occur [6]. While cross-reactivity with *A. vasorum* has been shown to occur with HW Ag tests (with and without HT), this parasite is not yet known to occur in Florida, USA [20, 30, 36], but has been detected in North America. Very different positive predictive values for both HT and non-HT Ag tests could potentially be obtained if our study was repeated in countries where the prevalence of parasites such as *D. repens* or *A.vasorum* was different from the USA.

Regarding the effect of HT on HW Ag test results in dogs infected with *A. reconditum,* Gruntmeir et al. [14] evaluated HW Ag pre- and post-HT in dogs with necropsy-confirmed *D. immitis* infections and Mf identification. In this study, dogs were considered heartworm-positive if immature, female, and/or male filariae were detected in the heart, pulmonary arteries, or thoracic cavity. Of the 58 heartworm-infected dogs, 12 had both *D. immitis* and *A. reconditum* Mf and 2 had only *A. reconditum* Mf. The two heartworm-infected dogs with only *A. reconditum* Mf converted from HW Ag negative to positive post-HT. If these were field collected samples without necropsy data, it would be impossible to determine whether these dogs were coinfected with HW or if these were false-negative results after HT. In this same study, all 105 heartworm-uninfected dogs were HW Ag negative pre- and post-HT, including 8 dogs with *A. reconditum* Mf. According to the study results, it is unlikely that HT would result in *A. reconditum* infections being falsely identified as HW infections; however, data from dogs with necropsy confirmation of exclusively *A. reconditum* infections would make this study more robust. In our study, the four dogs with *A. reconditum* Mf were all HW Ag-positive post-HT. Based on the Gruntmeir [14] study, coinfections with *D. immitis* and *A. reconditum* do occur in dogs in Florida, USA, as in that study, of 22 dogs with *A. reconditum* Mf, 14 were coinfected with *D. immitis*. Importantly, most published field studies have demonstrated that the majority of dogs with *A. reconditum* Mf did not convert from HW negative to positive post-HT [9, 23].

There are just two case reports that suggest false-positive HW Ag test results in dogs infected with *Acanthocheilonema dracunculoides* [27, 31]. In one of these studies, it was confirmed by necropsy that *D. immitis* were absent, but there were many *A. dracunculoides* adults, with massive accompanying microfilaremia. This dog was HW Ag-positive without HT. Overall, these case reports suggest that if there is a high burden of subcutaneous *A. dracunculoides* infection, there could be high concentrations of antigen in circulation from the adult worms and/or Mf, which could be detected by HW tests, with or without HT. It is important to note that *A. dracunculoides* is not known to occur in the USA and was not detected by PCR in our study. Other parasites such as *Spirocerca lupi* have also been associated with false-positive HW Ag tests. *Spirocerca lupi* is considered rare in dogs in the region, although infections have previously been described [1].

The AHS recommends that HT could be considered if there is a negative HW antigen test, but Mf are detected and/or the clinical presentation is consistent with heartworm disease. Our study evaluated how the total number of HW-positive dogs would increase if they were screened using HT (against current AHS recommendations). The proportion of positive HW Ag test results increased from 14% or 16% with POC and microtiter well assays, respectively, to 28% after HT serum was used. Previous studies have reported HW Ag status is more likely to change from negative to positive after HT in shelter dogs than in pet dogs [23], and shelter dogs are a higher risk population for *D. immitis* infection. Some of the dogs identified only on HT Ag testing in our study may have had prepatent infections with immature adult worms, low worm burdens, or immune complexes that blocked Ag detection [5, 13, 23]. A study using necropsy confirmation to evaluate the effect of HT on the sensitivity and specificity of DiroCHEK® in any type of HW infection (immature adult, male only, female only, or a combination) in stray dogs in north central Florida (*n* = 248) demonstrated that HT increased the sensitivity from 86.9 to 94.6% at little cost to specificity (from 97.8 to 96.1%; [13]. HT detected additional dogs with low worm burdens, all male, and immature infections, and also detected all dogs with worm burdens of 11–20 and 21–40, whereas non-HT missed two of these cases (4.3% and 3.5% of the 11–20 and 21–40 worm burden groups, respectively). In our study, amicrofilaraemic dogs that were only detected as HW positive post-HT could have had occult HW infections or false-positive results. In a recently published study by Gruntmeir et al. [14], 13/28 HW-infected, amicrofilaremic dogs were HW Ag-positive pre-HT and 8 additional dogs became positive post-HT. These data suggest that amcirofilaremic dogs in our study that converted to a positive Ag test post-HT could have been infected with heartworms.

If HT is used when screening shelter populations, the increased cost of testing and delay in obtaining results could render this method impractical if test results are time-sensitive and finances are limited, as is often the case when rehoming or rescuing dogs. Current HT methodology heats serum to 103 °C using a dry heat block followed by centrifugation at 16,000×*g* (faster than a typical clinic centrifuge) and is usually performed in a reference laboratory [23].

The AHS recommends that both non-HT Ag and Mf testing should be used for routine HW screening in dogs. In a shelter setting, the fastest and most cost-effective protocol is to perform POC Ag HW tests and wet mounts. In our population, six dogs were POC Ag HW negative and Mf positive. However, further laboratory testing revealed that only two of the six dogs were infected with *D. immitis* Mf, emphasizing the importance of carefully evaluating Mf to confirm that they are *D. immitis* before proceeding to treatment (https://capcvet.org/guidelines/heartworm/). If there is limited time and skilled personnel to perform Mf testing, as in many shelters, canine screening using POC Ag tests alone might preserve resources while only minimally compromising diagnostic sensitivity; adopters could also be advised to follow up with their local veterinarian for microfilaria testing.

Although the Ab testing methods used in our study are not clinically validated for use in dogs, there were some interesting general agreements between Ab test results and other methods. For example, 84.6% of dogs that had *D. immitis* Mf were Ab positive compared to 25.3% of dogs that either had no Mf or other Mf species (Table 4). Of dogs that were HW Ag-negative by DiroCHEK®, either with or without heat treatment, not more than 25% were Ab-positive and > 67.9% of dogs that were Ab-positive were also DiroCHEK®-positive, with or without heat treatment. In a previous study evaluating Ab testing in dogs, antibodies were detected as early as 11 weeks after experimental infection, before Ag could be detected using currently available tests [18]. A validated test to detect dogs with larval prepatent infections could have clinical utility. If accurate, the test could identify dogs with potentially early HW infections. Coupled with a negative Ag test, these dogs could be promptly placed on a heartworm preventive and then monitored over time with heat-treated Ag testing and Mf testing to determine whether the infection developed to patency.

Limitations of this study included a relatively small sample size that resulted in large confidence intervals for test performance estimates. Although all dogs included in the study were identified as unowned strays at the time of intake and testing, it is possible that some had received HW preventive medications prior to shelter admission that could have affected test results. The dogs enrolled in the study were predominantly young, reflecting the typical age distribution of stray dogs in this region. This may have selected for dogs with relatively low total lifetime exposure to HW, which could have an unknown effect in test performance. Necropsy has traditionally been considered the ideal comparator for confirmation of HW infection status, but was not performed in this population of dogs. Therefore, it is likely that the infection status of some dogs was incorrectly categorized based on the results of the tests performed.

## Conclusions

In this shelter-based canine study, routine POC HW Ag and Mf tests were negative in approximately half the dogs that were detected as HW positive when HT of sera was performed prior to testing. Adding Mf detection methods to POC Ag testing, as is currently recommended, only marginally increased detection of positive dogs.

Given the substantial increase in dogs detected as positive with DiroCHECK HT compared to POC testing in our study, HT HW Ag testing alone could be considered if the priority is to minimize the proportion of dogs incorrectly classified as HW-negative in animal shelters while using the minimum number of test modalities. However, heat treatment may also decrease the specificity of HW Ag tests slightly, which would result in some dogs being incorrectly classified as HW-positive [13]. Understanding the prevalence of parasites that are more likely to become detected post-HT, such as *D. repens* and *A. vasorum,* is important before implementing HT in a screening protocol; international differences in prevalence could change resultant diagnostic statistics substantially, depending on where the study was performed. Our identification of a stray dog in the US with *D. repens* microfilariae is concerning. Without knowing the origin of this Husky mixed breed dog, we cannot know if this is an autochthonous case or imported case. In a related study [15], we also identified a stray domestic shorthair cat with *D. repens* microfilariae (1,099 Mf/ml). Both of the infected animals came from the same south Florida shelter. If *D. repens* is an emerging parasite in this region, or other regions of the US, this could have implications for the use of HT HW Ag testing.

There is a need to improve access to highly accurate, rapid, and inexpensive large-scale HW testing for dogs in animal shelters. Developing validated and practical protocols that incorporate heat or chemical treatment to disrupt immune complexes that can interfere with POC or well-based ELISA HW Ag tests, or decreasing the cost and time required for such testing in reference laboratories, might provide a solution to this unmet need. In light of these limitations in shelter settings, veterinarians examining newly adopted dogs with previously negative HW test results may consider additional testing, including Mf and/or HT HW Ag testing, and recommend repeat testing 6 months later to increase detection rates.

## Data Availability

The datasets during and/or analysed during the current study available from the corresponding author on reasonable request.

## Abbreviations

*A. reconditum*: *Acanthocheilonema reconditum*
Ab: Antibody
ANT: Antech heartworm antibody test
Ag: Antigen
CI: Confidence intervals
*D. immitis*: *Dirofilaria immitis*
DHW: DiroCHEK®
HT: Heat-treated antigen test
HW: Heartworm
ID: Identification
Mf: Microfilariae
MKT: Modified Knott’s test for *D. immitis*
−: Negative
N/A: Not applicable
+: Positive
Quantitative MKT: Microfilaria/ml using MKT
SNAP: IDEXX SNAP® Heartworm RT
SSA: HESKA SoloStep® FH
WM: Wet mount
WHW: WITNESS® Heartworm
